# *In vitro* assessment for primary stability of self-drilling orthodontic mini-implants: Pull-out testing at different insertion angles

**DOI:** 10.6026/973206300222792

**Published:** 2026-05-31

**Authors:** Poulomi Roy, Riddhi Chawla, Ashish Kumar, Harshit Pandya, Shikha Rastogi Gupta, Kashyap Shah

**Affiliations:** 1 Department of Orthodontics and dentofacial orthopaedics, Burdwan Dental College and Hospital, Burdwan, West Bengal, India; 2 Department of Dentistry, School of Dentistry, Central Asian University, Uzbekistan; 3 Department of Orthodontics and Dentofacial Orthopaedics, NIMS Dental College & Hospital, NIMS University, Rajasthan, Jaipur, India; 4 Department of Orthodontics and Dentofacial Orthopaedics, Geetanjali Dental College, Udaipur, India; 5 Department of Orthodontics and Dentofacial Orthopedics, Goenka Research Institute of Dental Science, Pethapur, Gandhinagar, Gujarat, India

**Keywords:** Orthodontic mini-implant, primary stability, insertion angle, pull-out test, temporary anchorage device (TAD)

## Abstract

Achieving optimal primary stability of orthodontic mini-implants remains a clinical challenge because the ideal insertion angle for self-drilling mini-implants has not been clearly established. Therefore, it is of interest to evaluate and compare the primary stability of self-drilling orthodontic mini-implants inserted into artificial bone blocks at four different angles (30°C, 45°C, 60°C and 90°C) using pull-out resistance testing. Eighty titanium alloy mini-implants (1.6 mm diameter, 8 mm length) were randomly allocated into four groups (n=20 each) and inserted into standardized synthetic bone blocks simulating D2 bone density with a custom angular guide, followed by pull-out testing in a universal testing machine at a crosshead speed of 1 mm/min. Mini-implants placed at 60°C showed the highest mean pull-out force (324.67 ±28.43 N), followed by 45°C (298.52 ±31.76 N), 90°C (267.38 ±25.91 N) and 30°C (231.14 ± 33.82 N), with statistically significant differences between the groups. Thus, insertion at 60°C to the bone surface provided the high primary stability. It may be considered the biomechanically most favorable angulation for self-drilling orthodontic mini-implants.

## Background:

This is because the introduction of temporary anchorage devices in the orthodontics practice has radically changed the capacity of treatment planning by giving clinicians the ability to have skeletal anchorage that is not dependent on patient compliance and dental unit cooperation [1]. Of the many types of temporary anchorage means, orthodontic mini-implants, also known as mini-screws or micro-implants, have received by far the highest levels of acceptance because of their small size, comparatively easy placement and removal process, instant loading, diverse clinical applications and good cost-effectiveness profile [2]. The devices are consistently used in a wide range of orthodontic mechanics such as en-masse retraction of anterior teeth, intrusion of overerupted molars, correcting canted occlusal planes, molar distalization and asymmetric tooth movement [3]. Although mini-implant failure has been widely used clinically, although generally successful, the failure rate of mini-implants still has been a consistent clinical complication with reports of failure rates ranging between 7 and 28 percent according to the study population, anatomical site, surgical procedure and loading regimen [4]. Mini-implant failure is largely explained as lack of primary stability during the insertion period and is the mechanical fixation obtained by direct connection between the screw threads and the bone tissue surrounding it before the onset of any biological healing process [5]. In contrast to conventional dental implants, which gain the capacity to be long-term stable due to the process of osseointegration, mini-implants are basically maintained in the organism through mechanical interlocking of the cortical bone, so the primary stability is the most important factor in determining the success of the treatment [6]. The primary stability of orthodontic mini-implants is a complex interaction of factors that may be more or less divided into the implant-related (diameter, length, thread design, surface characteristics), bone-related (cortical thickness, bone density, bone quality) and surgical technique factors (insertion method, pre-drilling protocol, insertion torque and insertion angle) [7]. The angle of insertion relative to the bone surface has been studied extensively among them since it directly dictates the level of cortical bone involvement, the actual thread-bone point of contact and the biomechanical connection between the provided orthodontic force vector and the mini-implant axis [8]. When using orthodontic mini-implants in the clinical practice, there are a number of practical and anatomical reasons why they may be placed at oblique angles versus being placed at right angles to the bone surface. Angulated insertion enhances the distance traversed by the path through the cortical plate maximizing cortical bone contact and is also likely to maximise mechanical retention [9]. The oblique insertion into the interradicular space also leads to the risks of dealing with root contact or damage being diminished and the presence of better accessibility in more anatomically restricted space as well as the ability to place the orthodontic force elements in a more desirable position in comparison to the occlusal plane [10]. The most frequently advised angles of insertion in the literature are between 30 to 90 degrees relative to bone surface with much options in clinical guideline and specialist recommendations [11]. The geometrical premise behind angulated insertion is that, the smaller the angle of insertion relative to the normal course of the teeth, the greater the apparent thickness of the cortical bone that the mini-implant penetrates, which depends on the cosecant value of the angle. In a 30°C angle, effective cortical path length is two times that at perpendicular insertion, however, [12]. Nonetheless, this geometric advantage should be counterbalanced with some possible biomechanical drawbacks, such as the concentration of stress on cortical bone entrance points, distortion of force distributions along the implant body and high bone micro-damage during insertion at extremely acute angles [13]. Some of the past studies have studied the connection between mini-implant stability and insertion angle by different methodological designs that include the use of finite element analysis, insertion and removal torque experiment, resonance frequencies and mechanical pull-out experiment. The overall result of finites element studies has indicated that the oblique insertion angles of 40°C-70°C are the best at stress distribution and help to boost retention [14]. These computational predictions have, however, not been consistent when experimentally tested by standardized mechanical testing, with some studies showing better results in perpendicular insertion [15] and other studies showing better results in oblique angle [16]. It is possible to explain the inconsistency in the existing literature by the fact that the methodology is largely heterogeneous (different bone substrate such as animal bone, cadaveric bone, synthetic bone), mini-implant specification, pre-drill protocols, the methodology of the test (pull-out test or push-out test or insertion/ removal torque) and the angle of experimentation [17]. Moreover, most studies have only compared insertion angles of two or three, thus they do not reveal the best angulation over a wider angle. It has been accepted that the systematic studies are needed to assess the multiple clinically relevant angles of insertion under the controlled conditions through the tested validated methodology [18]. The most common formulation of testing is considered to be pull-out testing as it is one of the most accurate and clinically significant tests in the evaluation of primary stability because it directly measures the amount of maximum axial force necessary to dislodge the implant in bone, which is a summation of the mechanical interlocking of the implant threads with the surrounding bone mass [19]. Therefore, it is of interest to evaluate the effect of different insertion angles on the primary stability of self-drilling orthodontic mini-implants using pull-out resistance testing.

## Materials and Methods:

## Study design:

This is an in vitro experimental research carried out under the biomechanics testing lab of the Faculty of Dentistry. The experiment was used to measure the pull-out resistance of self-drilling orthodontic mini-implant, which were inserted into various angles into a standardized synthetic bone block.

## Specifications of mini-implants:

In this study, self-drilling orthodontic mini-implants (n=80) which were commercially available were used. The mini-implants were of the same manufacturer and lot number to achieve dimensional uniformity.

Specifications were as shown:

[1] Material: Grade V titanium alloy (Ti-6Al-4V)

[2] Diameter: 1.6mm (thread outer diameter)

[3] Length: 8 mm (threaded portion)

[4] Thread pitch: 0.7 mm

[5] Thread design: Thread profile is single-lead buttress.

[6] Head design Bracket-type cross-slot head.

Prior to testing, all mini-implants were examined under a stereomicroscope (Leica S9i, Leica Microsystems, Germany) with a 10x magnification in order to observe structural integrity and lack of manufacturing defects. All specimens that had visible thread deformities, surface flaws and dimensional variation were rejected and substituted.

## Bone substrate:

The test substrate was made of standardized synthetic bone blocks (Sawbones, Pacific Research Laboratories, Vashon, WA, USA). The blocks were made up of a 2-mm layer of short fiber-filled epoxy resin which mimicked cortical bone (density: 0.80 g/cm^3^, compressive strength: 157 Mpa) bonded to a 30-mm layer of rigid polyurethane foam which mimicked cancellous bone (density: 0.32 g/cm^3^, cellular structure: 15 pcf). This was chosen so as to resemble bone quality of D2 which is more or less similar to the bone density that the mini-implants are usually placed. To provide uniform dimensions, all bone blocks were sliced to standardized rectangular bone blocks (40 mmx30 mmx32mm) using a low-speed precision saw. Before testing, specimens had been kept at room temperature (23 ± 2°C) and ambient levels of humidity.

## Group allocation:

The eighty mini-implants were then apportioned randomly into four groups of twenty specimens each of the respective designation of the angle of insertion as related to the surface of the cortical bone:

[1]Group A (n = 20): 30°C insertion angle

[2]Group B (n = 20): 45°C insertion angle

[3]Group C (n = 20): 60°C insertion angle

[4]Group D: 20 (n): 90°C insertion angle (perpendicular, control)

The selection bias was avoided by randomizing the mini-implants with the help of a computer-generated random number list, thus randomizing the mini-implants to groups.

## Custom angular guide to insertion:

Specially designed angular insertion guide was made in stainless steel so that it is easy to insert the light beam with the required accuracy upon the desired angle. The guide was made up of a base plate with a clamping mechanism to firmly hold the bone block and an adjustable guide sleeve, which could be locked under specific angular positions (30, 45, 60 and 90 degrees) with respect to the bone surface. The angular positions were adjusted to a digital inclinometer (Bosch GIM 60, Robert Bosch GmbH, Germany) with a tolerance of 0.1°C. The guide sleeve was internal diameter 1.7 mm which gave the mini-implant a tight guidance channel without limiting the self-drifting process.

## Mini-implant insertion protocol:

To remove the inter-operator variability, all mini-implants were placed by one trained operator. The bone block was firmly clamped in the insertion guide and the guide sleeve was positioned and fixed at the recommended angle. Drilling of pilot holes was not carried out because the mini-implants were self-drilling. The mini-implants were manually inserted through the guide sleeve at a controlled rate by the use of the manufacturer-provided hand driver. The insertion was continued until the entire threaded length (8 mm) was embedded in the bone block with the thread-to-smooth shaft transition at the level of the cortical bone surface. A digital torque gauge (Tohnichi BTG60CN-S, Tohnichi Manufacturing Co., Japan) with the hand driver was calibrated and used to measure the insertion torque of each specimen. The guide sleeve was then carefully removed after insertion and the angular guide dissected to reveal the specimen to be examined visually. Those with any apparent bone fracture, cortical plate rupture or poor seat were disqualified and substituted. A minimum distance of 10 mm was kept between the sites of the mini-implant insertion into the same bone block to avoid test sites interaction effects. Each bone block had a maximum of 3 sites of insertion.

## Pull-out testing protocol:

Pull-out test was conducted on the basis of a universal testing machine (Instron 5969, Instron Corp., Norwood, MA, USA), which had a 5 kN load cell. A purpose-designed pull-out testing device was made to fit the exact shape of the head of the mini-implants. This was a combination of a low clamping base to hold the bone block and a high gripping solution, which held the mini-implant head by a hook-like connection to the load cell using a flexible cable of steel. The pull-out force was made to act on the long axis of the mini-implant (axial pull-out) with a fixed crosshead speed of 1 mm/min until the point at which the mini-implant was fully disengaged with the bone block. The axial orientation of the pull out force was checked in relation to every specimen by aligning the bone block position in the set up with that at which the insertion angle took place such that the force was pulled on the axis of the implant and not on a fixed vertical axis. The force-displacement data were sampled at a rate of 100 Hz and the data were continuously recorded via an in-built data acquisition software (Bluehill Universal, Instron Corp.).

The force-displacement curves were used to obtain the following parameters:

[1]Maximum pull-out force (N): The force (maximum) measured in the pull-out test.

[2]Maximum force (mm): The maximum displacement at which peak force had taken place.

[3]Stiffness (N/mm): slope of the linear part of the force dislocation curve.

## Surface analysis:

After pull-out testing, 5 mini-implants of each group were randomly selected under a scanning electron microscope (JEOL JSM-6510LV, JEOL Ltd., Tokyo, Japan) at 50 and 200 magnifications to assess the characteristics of the thread surfaces and remainance of the bone material on the surface of the implants as a marker of the failure mode (interfacial failure or bone fracture).

## Statistical analysis:

All the data were registered and measured with the help of IBM SPSS statistics software (version 27.0, IBM Corp., Armonk, NY, USA). Each group was calculated using descriptive statistics, mean, standard deviation, median, minimum and maximum values. The Shapiro-Wilk test was used to determine the data normality and the Levene test was used to determine the homogeneity of the variances. One way analysis of variance (ANOVA) was used to compare the mean pull out force, displacement and stiffness of the four groups. Post-hoc pairwise differences were done with Tukey Honestly Significant Difference (HSD) test. The correlation between the pearson correlation analysis was used to determine the relationship between the pin insertion torque and the pull-out force. The statistical significance value was determined to be p < 0.05.

## Results:

All eighty mini-implants were successfully inserted and tested without specimen loss. No cortical plate fractures, thread stripping, or insertion-related damage were observed in any group. The mean insertion torque values increased with decreasing insertion angle, reflecting the greater cortical bone engagement associated with more oblique angulations (Table 1). The mean maximum pull-out force values and associated biomechanical parameters are presented in Table 2 One-way ANOVA revealed highly significant differences among the four groups for maximum pull-out force (F = 37.42, p < 0.001), displacement at maximum force (F = 8.17, p < 0.001) and stiffness (F = 11.93, p < 0.001). The 60°C insertion angle group demonstrated the highest mean pull-out force (324.67 ±28.43 N), followed by the 45°C group (298.52 ±31.76 N), the 90°C group (267.38 ±25.91 N) and the 30°C group (231.14 ±33.82 N). Tukey's HSD post-hoc analysis for maximum pull-out force revealed statistically significant differences between most group pairs, as detailed in Table 3 Pearson correlation analysis revealed a moderate positive correlation between insertion torque and pull-out force across the entire sample (r = 0.54, p < 0.001). When analyzed within individual groups, the correlation was significant within the 30°C group (r = 0.61, p = 0.004) and the 90°C group (r = 0.52, p = 0.019) but did not reach significance within the 45°C (r = 0.38, p = 0.098) and 60°C groups (r = 0.41, p = 0.072). Scanning electron microscopic examination revealed distinct patterns of bone residue distribution on the mini-implant surfaces after pull-out. Specimens from the 60°C group demonstrated the most uniform distribution of adherent synthetic bone material across the thread surfaces, indicating comprehensive thread engagement and predominantly interfacial shear failure. The 30°C group showed irregular patterns of bone residue concentrated primarily on the apical thread surfaces with evidence of cortical bone fragmentation near the entry point. The 90°C group exhibited relatively clean thread surfaces with less adherent bone material, suggesting lower thread engagement. The 45°C group displayed an intermediate pattern between the 60°C and 90°C groups.

## Discussion:

The current in vitro research was a systematic analysis of how the primary stability of self-drilling orthodontic mini-implants is affected by the angle of insertion via pull-out resistance tests. The findings clearly showed that the angle of insertion has a significant influence on the pull-out resistance such that the 60 angulation is the most stable in primary among the four angulations that were tested. The conclusions are positive rejection of the null hypothesis and biomechanical evidence to support the clinical suggestion of oblique insertion to enhance retention of mini-implants. The observation that the highest pull-out resistance was obtained with the 60o insertion angle is attributable to the interaction of a number of biomechanical variables. The more the insertion angle is less than perpendicular, the longer the effective path length through the cortical plate and the larger the cortical bone-to-thread contact area a nd the higher the mechanical interlocking [20]. The effective length of the cortical path at 60 is about 15 percent longer than in 90 (effective length= cortical thickness/sin 60) which corresponds to a significant increase in thread turns involving dense cortical bone. This increased cortical involvement is a direct contribution to increased resistance to axial pull-out forces because cortical bone makes the major mechanical contribution to mini-implant retention [21]. Nonetheless, the observation that further reduction of the insertion angle to 30 leveraged significantly reduced pull-out forces even though theoretically the path length of the cortices is larger indicates that there is an optimistic angular range beyond which further cortical activity is counter-productive. A number of pernicious biomechanical phenomena could describe this decrease in stability at extremely acute angles of insertion. At 30 degrees, the insertion of a mini-implant results in a very elliptical entry profile in the cortical plate and the effect of this is to focus the stresses on the cortical points of entry and exit of the trailing of the implant [22]. Such concentration of stress may cause micro-fracture and compressive fail of the cortical bone on the entry point and in virtuality subvert the very cortical anchorage that the oblique angle was designed to strengthen. Also, the component of the pull-out force perpendicular to the cortical plate surface grows in relation to the component of the force perpendicular to the cortical plate in acute angles which generates the possibility that the mini-implant leverages through the cortical bone as opposed to uniformly extracting [23]. This levering activity produces an effect of fulcrum at the cortical bone margin that induces local failure of bone in lower values of overall forces. This mechanism was supported by the findings of the present study in which SEM showed that cortical bone was fragmented on the specimen that was positioned on 30°C. The latter observations are consistent with earlier finite element studies that revealed that extremely acute insertion angles produce the highest stress concentrations at cortical bone margin which are stronger than the bone yield strength under lower applied loads than the moderate oblique angles [24]. The perpendicular 90°C insertion group had less pull-out resistance compared to the 60°C and 45°C degrees groups which is in line with less cortical bone engagement with the perpendicular placement. The mini-implant goes through the cortical plate at 90 degrees, passing through the shortest path possible and incorporating the least amount of thread turns through the dense cortical layer [25]. As compared to perpendicular insertion with all its merits of simplicity, lessened insertion challenge and more uniform stress distribution along the implant axis, this study biomechanical data indicates perpendicular insertion does not produce optimal primary stability. This observation is in line with other past studies that have revealed better retention of obliquely fitted mini-implants than perpendicularly f itted mini-implants [26]. The fact that the insertion torque values were all higher as the insertion angle became smaller agrees with the fact that a larger frictional resistance was experienced as the insertion followed longer paths in the cortical bone. Nevertheless, the correlation between the insertion torque and the pull-out force was moderate meaning that the insertion torque alone cannot be a good predictor of the primary stability at different angles of insertion. The clinical implications of this finding are significant because clinicians should not be led to believe that an increase in insertion torque is a sign of greater stability, especially at very acute angles where a high torque may represent an excessive compression and micro-damage of the bone instead of an increase in mechanical interlocking [27]. The values of stiffness which reflect the resistance to initial displacement of the bone-implant interface followed a similar pattern to pull-out force, with the highest stiffness observed in the 60 degree group. The clinical significance of this parameter is that orthodontic mini-implants are exposed to the sustained lateral forces in the course of the treatment and an increase in the interface stiffness implies more resistance to the first micromovement that could trigger progressive loosening [28]. This reduced stiffness of the 30 degree group, although with greater insertion torque, once again reinforces the fact that the quality of bone at the interface, which may be undermined by micro-damage caused by the insertion process at acute angles, is more significant than the sheer quantity of cortical involvement. The relationship between pull-out force and the displacement at maximum force was opposite, where the higher the angle of pull-out, the less displacement was measured, the greater the angle, the higher the displacement. This observation indicates that in acute angles, the failure mode entails gradual bone deformation and gradual thread disengagement throughout extended path of displacement which is in agreement with the more distributed cortical engagement geometry. Conversely, perpendicular insertion provides more sudden failure with less displacement, which represents a shorter and more focused zone of cortical involvement [29]. This study has both merits and demerits with respect to the use of standardized synthetic bone blocks. Synthetic bone substrates provide an excellent reproducibility and the biological variability of animal or cadaveric bone specimens is eliminated, thus permitting the isolation of the variable of interest precisely - which in this case is the insertion angle [30]. The synthetic bone structure that was employed in this research; 2-mm cortical layer over cancellous foam is similar to the buccal alveolar bone design found in typical sites used to place mini-implants. Nevertheless, artificial bone is unable to entirely recapitulate the heterogeneous microstructure, anisotropic mechanical properties, viscoelastic behavior and characteristics of biological response of living bone tissue [31]. The missing biological factors, including blood supply, cellular remodelling and inflammation response imply that the findings are those of mechanical primary stability and not the biological mechanisms involved in sustaining stability. Also, pull-out testing technology measures axial resistance to displacement and the clinical orthodontic forces are usually used in a lateral, tangential, or combined direction [32]. Although axial pull-out resistance has been shown to be a valid and repeatable measure of mechanical interlocking, the clinical loading environment is s ignificantly more complicated. It would be prudent to add the load in a lateral direction, fatigue experimentation in a cyclic mode and c ombined loading regimen in future studies to gain a clearer understanding of in-service biomechanical behaviour of angulated mini-implants [33]. The other limitation is the usage of one mini-implant design and dimension. There are varying thread designs, diameter and length that could be affected by the insertion angle to determine the primary stability. The results of this research must, however, be discussed in the context of the mini-implant specification that has been utilized and may not be readily applicable to all of the commercially available systems [34]. Also, such aspects of clinical practice as soft tissue thickness, closeness to the roots of teeth, differences in bone quality between patients and operator proficiency did not come into consideration in this controlled laboratory environment. Although these limitations are present, there is an evident clinical relevance of present findings. The fact that 60o was found as the best angle of insertion gives clinical practitioners an evidence-based guideline on how to position mini-implants. This angulation is a pragmatic setting which makes the most out of cortical bone involvement and it does not bring up the undesirable stress focus impacts of acute angles and it can be easily accomplished in most clinical placement locations within the maxillary and mandibular buccal alveolar bone [35].

## Conclusion:

The insertion angle significantly influenced the primary stability of self-drilling orthodontic mini-implants, confirming that angulation is an important determinant of mechanical retention. Mini-implants inserted at 60°C demonstrated the highest pull-out resistance and overall primary stability, while 45°C also showed favorable performance compared with 90°C and 30°C. Therefore, an insertion angle of approximately 60°C relative to the bone surface may be considered the most biomechanically favorable option for optimizing primary stability under the conditions of this *in vitro* study.

## Figures and Tables

**Table 1:** Insertion torque values (Ncm) by insertion angle group

**Group**	**Insertion angle**	**n**	**Mean ± SD (Ncm)**	**Minimum**	**Maximum**	**p-value (ANOVA)**
A	30°C	20	14.82 ± 2.64	10.3	19.7	
B	45°C	20	12.47 ± 2.31	8.6	17.2	< 0.001
C	60°C	20	10.93 ± 1.98	7.4	14.8	
D	90°C	20	8.56 ± 1.72	5.9	12.1	
Post-hoc comparisons
revealed statistically significant
differences between all group pairs
(p < 0.05) except between
Groups B and C (p = 0.067).

**Table 2:** Pull-out testing results by insertion angle group

**Group**	**Angle**	**n**	**Max Pull-Out Force (N) Mean ± SD**	**Displacement at Max Force (mm) Mean ± SD**	**Stiffness (N/mm) Mean ± SD**
A	30°C	20	231.14 ± 33.82	1.87 ± 0.34	156.23 ± 24.67
B	45°C	20	298.52 ± 31.76	1.64 ± 0.28	203.48 ± 29.14
C	60°C	20	324.67 ± 28.43	1.52 ± 0.23	231.72 ± 27.83
D	90°C	20	267.38 ± 25.91	1.43 ± 0.21	214.56 ± 22.47
			p < 0.001	p < 0.001	p < 0.001
All pairwise comparisons
demonstrated statistically significant
differences (p < 0.05).

**Table 3:** Post-hoc pairwise comparisons (Tukey's HSD) for maximum pull-out force

**Comparison**	**Mean Difference (N)**	**95% CI**	**p-value**
30°C vs. 45°C	-67.38	-92.14 to -42.62	< 0.001
30°C vs. 60°C	-93.53	-118.29 to -68.77	< 0.001
30°C vs. 90°C	-36.24	-61.00 to -11.48	0.002
45°C vs. 60°C	-26.15	-50.91 to -1.39	0.034
45°C vs. 90°C	31.14	6.38 to 55.90	0.008
60°C vs. 90°C	57.29	32.53 to 82.05	< 0.001

## References

[1] Sousa-Santos P (2025). Bioengineering (Basel)..

[2] Singh J (2021). J Pharm Bioallied Sci..

[3] Baser B, Ozel MB. (2024). Adv Clin Exp Med..

[4] Barros SE (2021). Prog Orthod..

[5] Puls GL (2024). Dental Press J Orthod..

[6] Sreenivasagan S (2021). J Contemp Dent Pract..

[7] Abdalla KL, Mahmood TMA. (2023). Heliyon..

[8] Ravi J (2023). Dental Press J Orthod..

[9] Romanec CL (2025). J Clin Med..

[10] Gezer P, Yilanci H. (2023). Int Orthod..

[11] Tatli U (2019). Am J Orthod Dentofacial Orthop..

[12] Duske K (2025). J Orofac Orthop..

[13] Assad-Loss TF (2017). Dental Press J Orthod..

[14] Sousa-Santos C (2024). Materials (Basel)..

[15] Mohan S (2022). J Orthod..

[16] Xavier J (2023). Contemp Clin Dent..

[17] Möhlhenrich SC (2021). Int J Oral Maxillofac Surg..

[18] Copello FM (2021). Dental Press J Orthod..

[19] Dal Paz J J (2025). J Clin Exp Dent..

[20] Tseng YC (2017). Kaohsiung J Med Sci..

[21] Hergel CA (2019). Eur Oral Res..

[22] Di Leonardo B (2018). J Orofac Orthop..

[23] Cunha AC (2017). Int J Oral Maxillofac Surg..

[24] Tepedino M (2017). Head Face Med..

[25] Popova NV (2021). Stomatologiia (Mosk)..

[26] Özkan S (2022). Am J Orthod Dentofacial Orthop..

[27] Giri M (2020). J World Fed Orthod..

[28] Migliorati M (2021). Orthod Craniofac Res..

[29] Vieira CA (2021). Acta Odontol Latinoam..

[30] Silva AS (2021). Int J Dent. 2021.

[31] Möhlhenrich SC (2019). Eur J Oral Sci..

[32] Jang TH (2018). Am J Orthod Dentofacial Orthop..

[33] Steiner C (2020). Int J Oral Maxillofac Implants..

[34] Selvaraj S (2024). Cureus..

[35] Walter A (2023). J Orthod..

